# The effect of daily consumption of probiotic yogurt on liver enzymes, steatosis and fibrosis in patients with nonalcoholic fatty liver disease (NAFLD): study protocol for a randomized clinical trial

**DOI:** 10.1186/s12876-022-02176-2

**Published:** 2022-03-07

**Authors:** Sara Ebrahimi-Mousavi, Seyed Moayed Alavian, Amir Ali Sohrabpour, Fatemeh Dashti, Kurosh Djafarian, Ahmad Esmaillzadeh

**Affiliations:** 1 Students’ Scientific Research Center, Tehran University of Medical Sciences, Tehran, Iran; 2 Department of Clinical Nutrition, School of Nutritional Sciences and Dietetics, Tehran University of Medical Sciences, Tehran, Iran; 3 Baqiyatallah Research Center for Gastroenterology and Liver Disease, Baqyiatallah University of Medical Sciences, Tehran, Iran; 4 The Liver, Pancreatic, and Biliary Disease Research Center, Digestive Disease Research Institute, Shariati Hospital, Tehran University of Medical Sciences, Tehran, Iran; 5 Department of Community Nutrition, School of Nutritional Sciences and Dietetics, Tehran University of Medical Sciences, P.O. Box 14155-6117, Tehran, Iran; 6 Obesity and Eating Habits Research Center, Endocrinology and Metabolism Molecular-Cellular Sciences Institute, Tehran University of Medical Sciences, Tehran, Iran; 7 Department of Community Nutrition, School of Nutrition and Food Science, Isfahan University of Medical Sciences, Isfahan, Iran

**Keywords:** NAFLD, Probiotic, Yogurt, Fatty liver, Steatosis, Fibrosis

## Abstract

**Background:**

Given the increasing prevalence of non-alcoholic fatty liver disease, it is necessary to find an easy and cost-effective method in its management and treatment. Probiotics are a group of living microorganisms that might affect NAFLD through the intestinal-liver axis. The present clinical trial aims to examine the effect of probiotic yogurt consumption on liver enzymes, steatosis and liver fibrosis in patients with NAFLD.

**Methods:**

Sixty-eight patients with NAFLD will be recruited in this study. After block matching for sex, BMI and age, patients will be randomly assigned to receive 300 g/d probiotic yogurt containing 10^6^ cfu/g of Lactobacillus acidophilus and Bifidobacterium lactis strains or 300 g/d plain yogurt daily for 12 weeks and those in the control group would receive similar amounts of plain yogurts. Weight, height, and waist circumference will be measured at study baseline and after the intervention. Biochemical indicators including plasma glucose, serum insulin, lipid profile, liver markers (ALT, AST and GGT) will be examined at study baseline and at the end of the trial. Insulin resistance and insulin sensitivity will be determined using the HOMA-IR and QUICKI equation. The degree of steatosis and hepatic fibrosis will also be assessed at the beginning and end of the intervention by the same gastroenterologist using elastography with fibroscan.

**Discussion:**

Probiotics have been suggested as a new strategy in the management of NAFLD. Their effects might be mediated through intestinal microbiota modification and production of short-chain fatty acids. Consumption of probiotic-enriched foods, rather than their supplements, might be a cost-effective method for long-term use in these patients. In case of finding the beneficial effects of probiotic yogurt consumption in the current clinical trial, its inclusion in the dietary plan of NAFLD patients can be recommended.

*Trial registration* This clinical trial was registered in Iranian Registry of Clinical Trials (www.irct.ir) at 2021-04-19 with code number of IRCT20210201050210N1.

## Background

Non-alcoholic fatty liver disease (NAFLD) is defined as an exacerbation of the triglyceride content in hepatocytes (greater than 5% of liver weight) without excessive alcohol consumption [1]. NAFLD is the most common type of chronic liver disease in the world which might result in simple steatosis, non-alcoholic steatohepatitis (NASH) and fibrosis. These complication can eventually progress to cirrhosis or liver carcinoma [2]. The general prevalence of NAFLD, based on ultrasound screening, is estimated to be 20–30% [3]. In Asia, it has been reported that 15 to 40% of population are affected [4]. According to a meta-analysis, 32% of population in the Middle East and 34% in Iran suffer from this condition [5].

Although weight loss has been the most effective strategy to manage this condition [6–8], dieting for weight loss has had little success in obese people in the long run. Therefore, it is necessary to find complementary therapies to control this condition. The composition of gut microbiota has recently gained great attention in several chronic diseases including fatty liver. Gut microbiota seem to affect this condition through various pathways including increased ethanol production, increased lipopolysaccharide (LPS) release and activating inflammatory cytokines [9, 10]. Therefore, modification of gut microbiota through probiotics might be a new strategy for effective management of NAFLD [11–13]. Earlier studies have shown the efficacy of probiotic supplements in improving levels of liver enzymes [14–17], lipid profiles [14, 18], inflammatory factors [19–21], steatosis [16] and fibrosis [16]. However, due to the high cost of supplements, low access and the possibility of inducing infection and inflammation by their high dosages in long time [14–17], taking probiotics in the context of foods might be a superior choice than supplements. Probiotic foods also contain other nutrients that can be effective in liver health. In addition, findings based on supplements of probiotics may not be generalizable to all patients because of their short-term high dosage use in clinical trials [22–26].

Despite several studies about the effect of probiotic supplements on liver health, limited investigations have assessed the effect of probiotic-enriched foods. In a clinical trial on 102 NAFLD patients, consumption of synbiotic yogurt for 24-week resulted in a significant reduction in liver enzymes and steatosis [27]. Nabavi et al., in a study on 62 patients with NAFLD, administered probiotic yogurt containing Lactobacillus acidophilus and Bifidobacterium for 12 weeks and found a significant reduction in liver fat and enzymes [28, 29]. None of these studies have examined the effect of such interventions on liver fibrosis. In addition, both studies have used ultrasound, rather than fibroscan, to examine liver fat, which might be subject to errors due to subjective decisions. Also, as evidenced in the latest systematic review and meta-analysis, further studies are needed to investigate the effect of probiotics on liver fibrosis, and since studies on probiotic foods are limited. Therefore, the aim of this study was to evaluate the effect of probiotic yogurt consumption on liver enzymes, steatosis and liver fibrosis in patients with NAFLD.

## Patients and methods

### Participants

This study will be a parallel randomized clinical trial (CRT) that will be conducted in Tehran, Iran, in 2021. The sample size was calculated by considering the type 1 error of 5%, the study power of 80% and the hepatic steatosis levels [30] (based on CAP) as a key variable based on data from previous studies. Considering the mean difference in hepatic steatosis levels of 2 dB/mL between the two groups [12], using the suggested formula for parallel clinical trials, we will need 28 patients in each group. However, by taking a possible drop-out of 20% into account, we will recruit 34 patients in each group based on the study inclusion criteria. Outpatients with NAFLD will be recruited from private clinics in Tehran, Iran. The diagnosis of NAFLD will be done by a gastroenterologist based on examination with a fiberoscan. The study is ethically approved by the Bioethics Committee of Tehran University of Medical Sciences, Tehran, Iran, and is registered in the Iranian Registry of Clinical Trials website (http://www.irct.ir).

### Inclusion criteria

In this study, we will recruit patients with NAFLD, whom condition was approved by a gastroenterologist using fiberoscan examination. Individuals aged 20–60 years with a body mass index between 25 to 35 kg/m^2^ that have a fixed plan for medication use during the last 3 months will be included in the current study.

### Non-inclusion criteria

We will not include smokers, those with a history of alcohol use, pregnant or lactating women or those planning to get pregnant in the next three months**,** individuals with pathologic conditions affecting the liver, including acute and chronic hepatitis, viral hepatitis, liver transplantation, autonomic hepatitis, hemochromatosis, primary biliary cirrhosis or Wilson's disease, antitrypsin deficiency, thyroid disease and lactose intolerance. In addition, patients who were taking antibiotics and medications affecting serum lipids as well as those taking multivitamin-minerals during the previous month will not be included. Use of any probiotic products during the last two months will be another non-inclusion criteria.

### Exclusion criteria

We will exclude patients who are on a specific diet throughout the study, those who are consuming alcohol and/or tobacco as well as individuals who are taking multivitamin-minerals throughout the study. In addition, any changes in the medications during the study will also be a criterion for exclusion.

### Study design and intervention

**Fig. 1:**
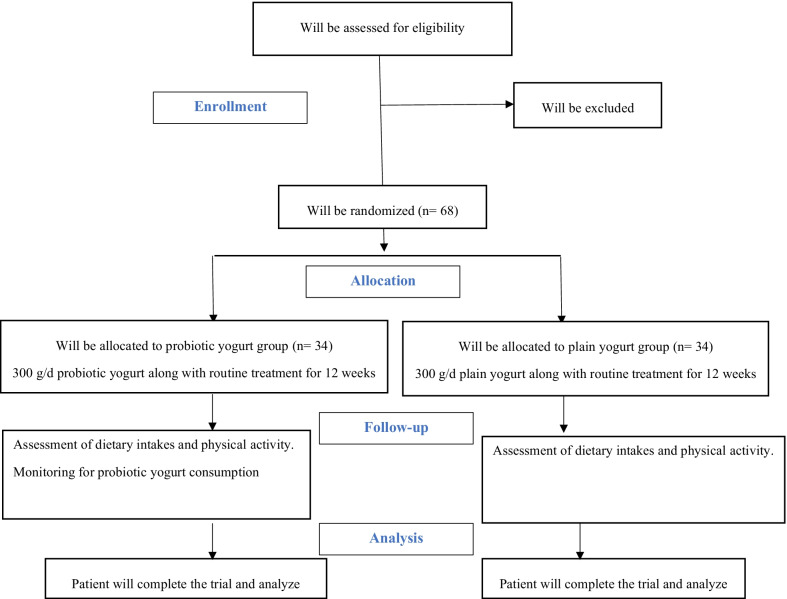
Diagram of the study design

**Fig. 2:**
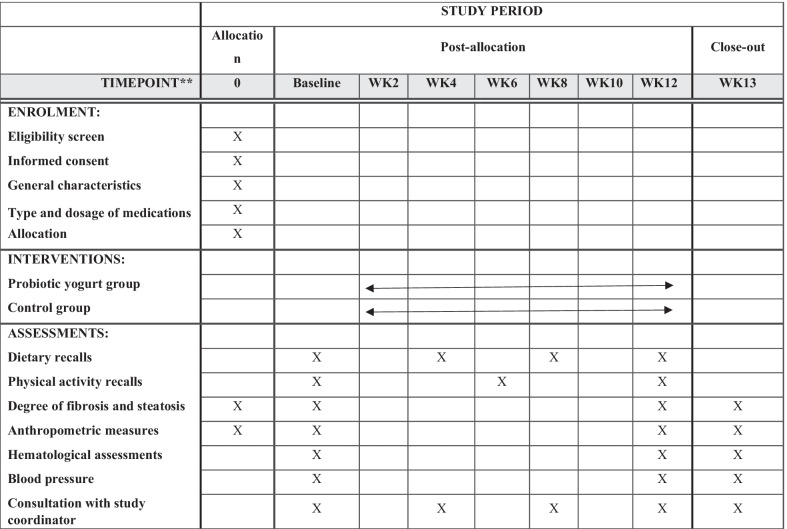
Flowchart of enrolment, intervention and assessments. NAFLD: none-alcohol fatty liver disease, WK: week. The “X” refers to time of allocation, intervention and assessment of variables

**Fig. 3:**
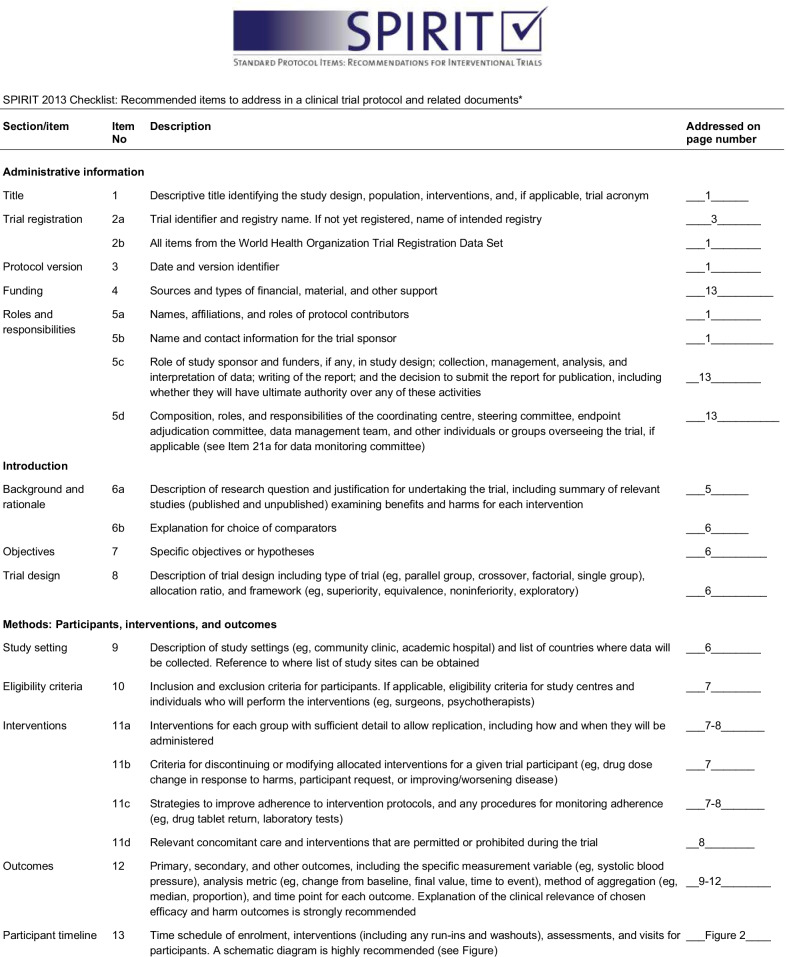

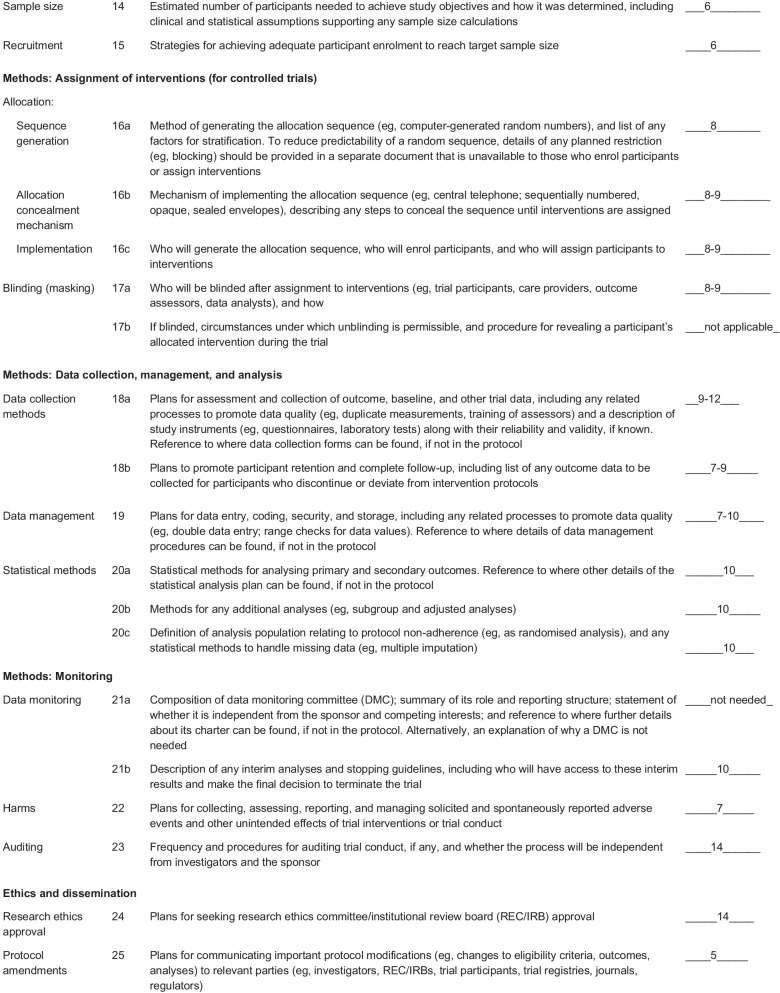

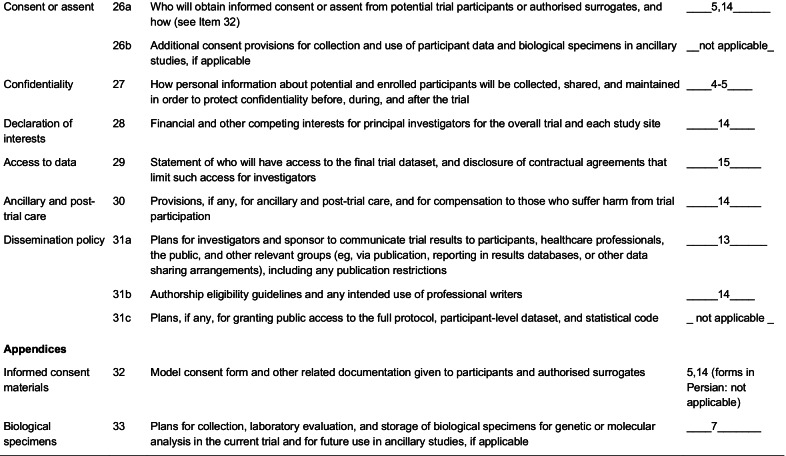
Standard Protocol Items: Recommendations for Interventional Trials (SPIRIT) chart of the enrollments and assessments during randomized controlled

A flowchart of the study design is shown Fig. 1. Flow-diagram of study process is shown in Fig. 2. In the first phase of this study, we will screen 300 patients with NAFLD based on the inclusion criteria mentioned above. Then, those who meet the criteria will be included in the study. All participants will be asked to complete and sign the written informed consent. Before the start of intervention, all study participants will be in a 2-week run-in period, during which four 24-h food recalls (two working days and one non-working day) will be used to collect dietary information. In addition, subjects will be requested to record their physical activity for two days (one working day and one weekend day) during this period. Further information about demographic characteristics, past medical history, medication use as well as socioeconomic status (SES) will be collected in this period through the use of a comprehensive questionnaire in a face-to-face interview. Anthropometric indices, blood pressure, liver enzymes, insulin resistance, hepatic steatosis, liver fibrosis and lipid profile will be evaluated at study baseline. Then study participants will be classified into blocks based on age (20–40/ 40–60 years old), sex (male, female) and BMI (25–30/ 30-35 kg/m^2^). Therefore, the size of each block will be 2 people with the same age, sex and BMI. These participants will then be randomly assigned into intervention and non-intervention groups. Randomization will be done by a third person, who is not aware of the study aims and groups. The first person (say A) in a block will be assigned into the intervention group and the second person (say B) in that block will be assigned into the control group. All participants, study personals and laboratory staffs will be blind to the intervention. Participants in the intervention group will consume 300 g/d of probiotic yogurt containing 10^6^ cfu/g of Lactobacillus acidophilus and Bifidobacterium lactis strains for 12 weeks, while participants in the control group will consume 300 g/d of plain yogurt daily (without any added probiotics) for 12 weeks. Yogurt packages will be distributed to participants every two weeks. The appearance, packaging and taste of yogurts will be the same. Quality control of these yogurts in terms of colony forming units of live bacteria and other nutritional values will be evaluated before the study. In order to comply with the ethical requirements in research, all patients will receive a weight loss diet after ending the study. Participants will be asked not to change their routine lifestyle, dietary habits, and medicines throughout the study. All study outcomes will be examined again at the study end. The study was designed according to the Standard Protocol Items: Recommendations for Interventional Trials (SPIRIT) 2013 shown in Fig. 3.

### Adherence

Individuals will be asked to record their daily intake of probiotic or conventional yogurt in a checklist given them by researchers. To increase compliance and prevent forgetting about daily consumption of probiotic or conventional yogurt, participants will receive short messages on their cell phones every day as a reminder. In addition, each time yogurt packages are delivered, patients will be requested to return empty packages of consumed ones. To ensure the consumption of yogurts by the study participants, and not their family members, additional packages of conventional yogurts will be given to them for probable family members’ use.

### Outcomes

The outcomes of the present clinical trial would be assessment of steatosis, fibrosis, serum liver enzyme levels including aspartate aminotransferase (AST), alanine aminotransferase (ALT), alkaline phosphatase (ALP), Gamma-glutamyl transferase (GGT). Systolic (SBP) and diastolic blood pressure (DBP), lipid profiles including high-density lipoprotein cholesterol (HDL-C), low-density lipoprotein cholesterol (LDL-C), total cholesterol (TC), serum triglycerides (TG) fasting blood sugar (FBS), fasting insulin and glucose hemostasis will also be considered. Weight and BMI will also be assessed. All these measurements will be assessed at study baseline and the end of trial.

### Dietary intake and physical activity assessment

In order to ensure lack of change in dietary intakes of participants during the study, we will collect dietary intake data through the use of a 24-h dietary recall every three weeks throughout the study. These dietary recalls will be distributed through the week days to cover two working days and two weekends. Due to COVID-19 pandemic, dietary recalls will be completed through phone calls, not by face-to-face method. In these phone calls, participants will be asked to report their dietary intakes in a preceding day based on household measures. All reported foods in these recalls will then be converted to grams using available booklets. The average intake based on four dietary recalls will be considered in the analysis.

Participants will be asked not to change their physical activity during the trial. To make sure about this, we will request study subjects to record their physical activity in four separate days throughout the study including two working days and two weekends. All participants will be instructed how to record the type, intensity and time spent in each activity in a certain form that will be given to them at the study baseline. In order to process the physical activity records, we will use earlier published guidelines about MET-h/day values for each type of physical activity, in which we will consider the time spent for a certain physical activity by each participant. All records would be reviewed immediately to find possible errors and the problems will be resolved by interview.

### Assessment of hepatic steatosis and fibrosis

Hepatic steatosis and fibrosis among all participants will be examined by transient elastography with fibroscan by the same gastroenterologist at the beginning and end of the study. Patients will be asked to lie down on their back and place the right hand above their head. Then the probe will be placed on the right side of the liver in patients’ intercostal area. The results of liver fibrosis will be expressed as Kilopascal (Kpa). The interpretation of fibrosis assessment results will be based on METAVIR Score [31]. The controlled attenuation parameter (CAP) test will be used to assess steatosis and the results will be reported in dB/m. CAP results will vary between 100 and 400 dB/m.

### Biochemical assessment

To assess the level of liver enzymes (ALT, AST, GGT), blood sugar and lipid profiles (including total cholesterol, serum triglyceride and HDL cholesterol), 10 cc of intravenous blood samples will be collected after 10–12 h of fasting. Blood samples are centrifuged for 10 min at 2606.8 G and 4 °C to separate serum samples. The level of ALT and AST enzymes will be determined by photometry (Pars Azmoun) proposed by the IFCC (International Federation of Clinical Chemistry) method. GGT levels will also be assessed using the calorimetric-kinetic method proposed by the IFCC. Fasting blood glucose (FBS) levels will be measured by glucose oxidase and fasting insulin levels will be measured by ELISA method. Serum concentrations of total cholesterol and triglycerides will be measured by laboratory methods using enzymes such as cholesterol esterase, cholesterol oxidase and glycerol phosphate oxidase, respectively, using standard kits. HDL-C will be measured after deposition of apolipoprotein-B, which contains lipoproteins with phosphotungstic acid. LDL-C will be calculated from the values of TC, TG, HDL_C by the formula of Friedwald [32].

### Glucose homeostasis assessment

Homeostatic Model Assessment of Insulin Resistance (HOMA) and Quantitative Insulin Sensitivity Check Index (QUICKI) will be calculated based on suggested formulas [33, 34]

### Blood pressure assessment

Systolic blood pressure (SBP) and diastolic blood pressure (DBP) will be measured after 5 min of rest with a mercury sphygmomanometer from the patient's right hand while sitting. Patients should not drink, exercise or smoke for one hour prior to blood pressure assessment. They must also have an empty bladder before measuring their blood pressure. Blood pressure will be taken twice, 5 min apart, while the patient is at rest. Systolic blood pressure is equivalent to the pressure measured after hearing the first korotkoff sound, and diastolic blood pressure is equivalent to the pressure measured when the korotkoff sound disappears. The mean of the two measurements will be considered as systolic and diastolic blood pressure.

### Anthropometric assessments

To assess height, the person must be standing and barefoot with an accuracy of 0.5 cm. Weight will be measured using a digital scale with a minimum of clothing and barefoot with an accuracy of 100 gr. Waist circumference will be measured using a tape measure in the middle of the distance between the super iliac bone and the last rib with an accuracy of 0.5 cm. BMI will also be obtained by dividing weight in kilograms by height squared in meters [35].

### Other variables assessments

A general questionnaire will be used to collect information about participants’ age, sex, marital status (single /married), education (yes/no), supplement use (yes/ no), medical history, tobacco use (non-smoker /smoking history/smoker), occupation, history of drug use.

### Statistical analysis

In this study, all analyses will be done using SPSS software version 26. Normal distribution of data will be assessed by the application of Kolmogorov–Smirnov test. In case of a non-normal distribution, we will apply logarithmic transformation. Results will be reported as mean ± standard deviations (SD) or medians (interquartile ranges). General characteristics and dietary intake of individuals between the two groups will be compared using independent t-test and chi-square test as needed. Repeated measure analysis of variance (RM-ANOVA) will be used to examine the effects of probiotic yogurt consumption on outcome variables, where we will examine the effects of time, intervention as well as time-group interaction effects. In this analysis, baseline values of outcome variables will be controlled for. We will also perform post hoc tests after ANOVA analysis to examine the variables between study groups. In case of any difference between the two groups in terms of dietary intakes (other than the intervention) and physical activity throughout the study, these differences will be adjusted for in the analysis to reach an independent effect of intervention on study outcomes. Statistical significance will be set to 0.05.

### Ethics and dissemination

This clinical trial study was confirmed by the ethics committee of Tehran University of Medical Sciences (code: IR.TUMS.MEDICINE.REC.1399.1006). Furthermore, it was registered in the Iranian Registry of Clinical Trials (www.irct.ir) at 2021-04-19 (code: IRCT20210201050210N1).

### Trial status

The recruitment started on December 2021.

## Discussion

NAFLD is an important public health issue due to its high global prevalence [31]. It is a clinical syndrome from simple steatosis to cirrhosis, which is associated with insulin resistance, lipo-toxicity, inflammation and obesity [31]. Changes in the composition of gut microbiota, called dysbiosis, are strongly associated with the development and progression of NAFLD [31]. Probiotics play an important role in modulating intestinal bacterial flora and modifying intestinal permeability [31]. Furthermore, the production of short-chain fatty acids by probiotics during fermentation can affect inflammatory, antioxidant, and gene expression pathways, through which they might prevent the progression of liver damage [31]. Previous investigations have reported that consuming probiotic yogurt improves lipid profile, glycemic variables, liver enzyme levels, and steatosis in patients with NAFLD. We are aware of no study examining the effect of probiotic yogurt on liver fibrosis [31]. In addition, earlier studies have mostly applied ultrasound, which is a less accurate method than the fiber scan, to estimate liver fat. Therefore, the aim of our study would be to evaluate the effect of probiotic yogurt consumption on liver enzymes, steatosis and liver fibrosis in patients with NAFLD. Through this investigation and in case of finding an appropriate effect, we would introduce an efficient and cost-effective method to take benefit of probiotics in patients with fatty liver.

Despite several strengths of our study protocol including stratified permuted block randomization and double-blind, placebo-controlled design and conducting the study on newly diagnosed NAFLD patients by fibroscan, some limitations must also be taken into account: We will examine the compliance in this study through collecting dietary intake information by questionnaires. Due to limited funding, we are unable to examine gut microbiota and serum trimethylamine N-oxide (TMAO) as a biomarker for assessment of compliance in this study. We also use probiotic strains available in the food market and did not focus on specific strains that might have different effects. Future studies should examine the effect of foods fortified with specific probiotic strains on steatosis and fibrosis in patients with NAFLD. In addition, another concern might be a limited duration of intervention in this study which would be for 12 weeks. Although one might assume this time period too short for our intervention to affect liver fibrosis, other studies have applied almost the same duration for assessing the effect of intervention of fibrosis among patients with NAFLD.

## Data Availability

Not applicable.

## Abbreviations

NAFLD: Non-alcoholic fatty liver disease
NASH: Non-alcoholic steatohepatitis
ALT: Alanine aminotransferase
AST: Aspartate aminotransferase
GGT: Gamma-glutamyl transferase
BMI: Body mass index
ELISA: Enzyme-linked immunosorbent assay
HDL: High-density lipoprotein
LDL: Low-density lipoprotein
METs: Metabolic equivalents
HOMA-IR: Homeostatic Model Assessment for Insulin Resistance
QUICKI: Quantitative Insulin Sensitivity Check Index
RCT: Randomized clinical trial
SD: Standard deviation
SES: Socioeconomic status
TC: Total cholesterol
TG: Triglyceride
WC: Waist circumference
CAP: Controlled attenuation parameter
TMAO: Trimethylamine N-oxide
